# Open and compressed conformations of *Francisella tularensis* ClpP

**DOI:** 10.1002/prot.25197

**Published:** 2016-11-20

**Authors:** Laura Díaz‐Sáez, Genady Pankov, William N. Hunter

**Affiliations:** ^1^ Division of Biological Chemistry and Drug Discovery, School of Life SciencesUniversity of DundeeDundeeUnited Kingdom

**Keywords:** protease, active site, conformational change, X‐ray crystallography

## Abstract

Caseinolytic proteases are large oligomeric assemblies responsible for maintaining protein homeostasis in bacteria and in so doing influence a wide range of biological processes. The functional assembly involves three chaperones together with the oligomeric caseinolytic protease catalytic subunit P (ClpP). This protease represents a potential target for therapeutic intervention in pathogenic bacteria. Here, we detail an efficient protocol for production of recombinant ClpP from *Francisella tularensis*, and the structural characterization of three crystal forms which grow under similar conditions. One crystal form reveals a compressed state of the ClpP tetradecamer and two forms an open state. A comparison of the two types of structure infers that differences at the enzyme active site result from a conformational change involving a highly localized disorder‐order transition of a β‐strand α‐helix combination. This transition occurs at a subunit‐subunit interface. Our study may now underpin future efforts in a structure‐based approach to target ClpP for inhibitor or activator development. Proteins 2016; 85:188–194. © 2016 Wiley Periodicals, Inc.

**Abbreviations**
AAA: ATPases Associated with diverse cellular Activities
ASA: accessible surface area

## INTRODUCTION

The ATP‐dependent caseinolytic proteases (Clp) maintain protein homeostasis in bacteria, especially under conditions of stress.^1^ These proteases, by virtue of regulating protein levels, have pleiotropic effects and indirectly modulate gene expression, cell motility and division, the removal of stress‐damaged proteins and are also implicated in pathogenesis by promoting virulence factor expression.^1^, ^2^

The functional Clp protease is a large multi‐protein complex, with an assembly of 14 subunits of the promiscuous serine protease ClpP at the core. This ClpP tetradecamer, a dimer of heptamers each arranged in a ring, displays an overall cylindrical shape forming an internal chamber where proteolysis of unfolded proteins occurs. The catalytic core is surrounded by accessory proteins, AAA^+^ (ATPases Associated with diverse cellular Activities) chaperones termed ClpA, ClpX and ClpC, that regulate the presentation of substrates into the active sites.^1^, ^3^ Two axial pores are formed by the complex and these allow peptide substrates access to the active sites when an open conformation is present.^4^ The products are released through pores in the equatorial plane that are generated after a conformational change to a compressed state.^4^, ^5^ ClpP, which is highly conserved across species, has been the subject of a number of crystallographic studies.^6^ The open and compressed conformations of ClpP have been characterized for the enzymes from *Bacillus subtilis* (*Bs*ClpP),^5^ *Mycobacterium tuberculosis,* 7 and *Staphylococcus aureus*.^4^, ^8^

Clp proteases have been investigated as antibacterial drug targets and some inhibitors do appear to attenuate the virulence of *S. aureus* 9 and *Listera monocytogenes*.^10^ In addition, compounds that activate the complex can enhance unspecific proteolysis and therefore, also have the potential to act as antibiotics.^11^ We have identified ClpP as a potential drug target in the facultative intracellular bacterium *Francisella tularensis*, based on similar considerations applied to targets in *Pseudomonas aeruginosa*.^12^ *F. tularensis* is a highly virulent Gram‐negative aerobe classified as a category (A) bio‐warfare agent by the Centers for Disease Control and Prevention (http://www.cdc.gov). We initiated a study of *F. tularensis* ClpP (*Ft*ClpP) to lay the foundation for early stage drug discovery^13^ targeting this enzyme. A structure of apo‐*Ft*ClpP, in an open conformation at a resolution of 2.3 Å, was available in the Protein Data Bank (code: 3P2L) but there is no associated publication and only limited experimental details are available. Here we present the efficient production of a recombinant source of *Ft*ClpP, the crystal structure of a compressed form of the enzyme at 2.8 Å resolution and two crystal forms that reveal the structure of an open state at 1.9 and 1.7 Å resolution.

PDB codes: *Ft*ClpP:I 5G1Q, *Ft*ClpP:II 5G1R and *Ft*ClpP:III 5G1S.

## MATERIALS AND METHODS

### Recombinant protein production

Synthetic DNA, codon optimized for expression in *Escherichia coli* K12, encoding *Ft*ClpP (UniProt: Q5NH47) was purchased (GeneWiz). This gene was cloned into a modified pET15b vector (Novagen) that adds a hexahistidine tag (His_6_‐tag) followed by a TEV (tobacco etch virus) protease cleavage site at the N‐terminus of the product. The restriction enzymes used were BamHI (at the 5' end) and NdeI (at the 3' end of the DNA strand). The construct was sequenced as a check. Recombinant expression was carried out in *E. coli* BL21(DE3). The bacteria were cultured in 1 L of LB broth containing 50 μg mL^−1^ carbenicillin at 37°C until an O.D. of 0.6 at λ = 600 nm was achieved then the temperature was decreased to 20°C. Gene expression was induced by adding 1 m*M* IPTG and continued overnight. Cultures were centrifuged (4,000 *g* at 4°C for 10 minutes) to harvest the cells. The sample was then resuspended in buffer A (20 m*M* Tris‐HCl pH 7.4 and 200 m*M* NaCl) containing an EDTA‐free protease inhibitor cocktail tablet (Roche). Cell disruption was carried out using a French press (20 psi) followed by centrifugation (40,000*g* for 40 minutes at 4°C). The soluble proteins were loaded on a 5 mL HisTrap HP column (GE Healthcare), loaded with Ni^2+^, to perform affinity chromatography. The buffers employed for *Ft*ClpP purification were buffer A and buffer B (buffer A with 0.8*M* imidazole). A fraction at 20% buffer B contained the desired product *Ft*ClpP. The sample was incubated with His_6_‐tagged TEV protease (1 mg per 20 mg protein) at 4°C overnight while performing dialysis into buffer A to remove the imidazole. A second affinity purification step was then performed under the same conditions but this time to separate the His_6_‐tag and TEV protease from the desired product. Size exclusion chromatography was then pursued with *Ft*ClpP with a HiLoad 16/60 superdex 200 prep grade column (GE Healthcare) and buffer A. The yield was 10 mg *Ft*ClpP per litre of bacterial culture and a high level of protein purity was confirmed using SDS‐PAGE and MALDI‐TOF‐MS analyses. Protein concentration was determined using the predicted *ε*(*Ft*ClpP) = 8940 *M* ^−1^ cm^−1^ calculated with PROTPARAM.^14^

### Crystallization

*Ft*ClpP samples were concentrated to 20 mg mL^−1^ and various commercially available crystallization conditions were tested using 1:1 and 1:2 protein solution to reservoir ratios (final drop volumes of 0.2 and 0.3 μL, respectively) in sitting drop 96‐well plates. Orthorhombic crystals were obtained from The Classics Suite (Molecular Dimensions) condition B4 (0.2*M* NaCl, 30% v/v MPD (2‐methyl‐2,4‐pentanediol) and 0.1*M* sodium acetate pH 4.6) and optimized using 1:1 protein solution to reservoir ratio in a final drop volume of 4 μL with the hanging drop method.

### Crystallographic analyses

Crystals were flash‐cooled directly from drops and diffracted to 2.8 Å resolution in‐house. These samples were stored for use at the synchrotron. In one case, data were collected in‐house using a Rigaku M007HF X‐ray generator with a Saturn 944HG+ CCD detector, a 10° θ offset to compensate for a large mosaic spread of 0.9°, 10 seconds of exposure per 0.5° oscillation covering 115°. Data integration and analysis were carried out using iMOSFLM^15^ and AIMLESS.^16^ The crystal displayed space group *P*2_1_2_1_2 and unit cell dimensions of *a* = 113.5, *b* = 125.9, *c* = 96.95 Å. The Matthews coefficient calculated with the CCP4i suite^17^ suggested seven monomers in the asymmetric unit (*V*_m_ 2.2 Å^3^ Da^−1^ and bulk solvent about 45%). The *Ft*ClpP structure with PDB code 3P2L (2.3 Å resolution, space group *P*2_1_2_1_2 and unit cell dimensions *a* = 120.52, *b* = 128.82, *c* = 98.03 Å), also with seven subunits in the asymmetric unit, provided a model of a single subunit, without residues 128–149, for molecular replacement calculations in PhaserMR.^18^ This structure is termed *Ft*CLpP:I and represents the closed or compressed form of the tetradecamer assembly. Diffraction data were also collected at ESRF beamline BM30‐A from two samples, both of which displayed low mosaic spread (0.2°). High‐resolution data were processed and analyzed using XDS^19^ and AIMLESS. These crystal forms displayed similar morphology and the same orthorhombic symmetry, space group (*P*2_1_2_1_2 or an alternative setting) but two distinct forms are evident with unit cell dimensions of *a* = 117.90, *b* = 128.20, *c* = 98.26 Å and *a* = 98.49, *b* = 128.23, *c* = 355.18. These structures are named *Ft*ClpP:II and *Ft*ClpP:III and represent open forms of the protein assembly. The Matthews coefficients (*V*_m_ of 2.4 Å^3^ Da^−1^ and bulk solvent about 50%) suggested the presence of 7 and 21 subunits in the asymmetric units in *Ft*ClpP:II and *Ft*ClpP:III, respectively. *Ft*ClpP:II was solved using PhaserMR with subunit A from the 3P2L model in a similar fashion as employed for *Ft*ClpP:I. In the case of *Ft*ClpP:III, the search model was the refined *Ft*ClpP:II heptamer. The general refinement procedure that was applied in all three cases started with a round of rigid body refinement using REFMAC^20^ followed by cycles of restrained refinement. Electron density and difference maps were inspected, and modifications to the models were made with COOT.^21^ Refinement of *Ft*ClpP:I was terminated when we were satisfied that the reliable parts of the polypeptide had been modeled and that disordered residues were excluded. No attempts were made to include solvent in the refinement given the modest resolution. In the case of the high resolution *Ft*ClpP:II and III structures the refinement extended to including rotamers, water molecules and ligands. Tight non‐crystallographic symmetry restraints were used at the beginning of all refinements then removed gradually. The refinements were ended when inspection of the maps suggested no more changes were justified, and no significant changes in the *R*_free_ and *R*_work_ were observed. Crystallographic statistics are presented in Table 1.

**Table 1:** Crystallographic Statistics

Structure	*Ft*ClpP:I	*Ft*ClpP:II	*Ft*ClpP:III
PDB code	5G1Q	5G1R	5G1S
Wavelength (Å)	1.5418	0.9799	0.9799
Space group	*P*2_1_2_1_2	*P*2_1_2_1_2	*P*22_1_2_1_
Unit cell dimension *a*, *b*, *c* (Å)	113.5 125.9 96.9	117.9 128.2 98.3	98.2 127.9 353.8
Resolution range**^**a**^** (Å)	63.61–2.84	47.04–1.90	48.80–1.70
No. reflections	90264	838386	2604010
Unique reflections	32741	117661	460688
Completeness (%)	97.8 (98.8)	100 (100)	94.8 (70.2)
*R*_merge_ **^**b**^**	0.098 (0.344)	0.085 (0.460)	0.084 (0.423)
Redundancy	2.8 (2.0)	7.1 (7.1)	5.7(2.5)
<*I*/*σ*(*I*)>	5.9 (2.2)	18.5 (4.4)	9.3 (1.4)
Wilson *B* (Å^2^)	15.6	10.8	9.8
*R*_work_ **^**c**^**/*R*_free_ **^**d**^**	0.249/0.326	0.201/0.269	0.204/0.242
*R*_free_ reflections (%)	4.99	4.98	4.98
DPI**^**e**^** (Å)	0.52	0.17	0.13
R.m.s.d. bond lengths (Å)/angles (º)	0.0079/1.243	0.018/1.875	0.008/1.285
Protein atoms/residues (Average *B*‐factors, Å^2^)	9337/1217 (37.6)	11663/2423 (20.9)	33173/8031 (14.6)
Water molecules (Average *B*‐factors, Å^2^)	–	1085 (25.8)	4011 (23.8)
Ligands (Average *B*‐factors, Å^2^)	–	5 MPD (47.1)	32 MPD (40.1)
		7 acetate (37.7)	21 acetate (24.5)
Ramachandran analyses			
Outliers (%)	0.8	0.2	0.04
Favoured regions (%)	93	96.9	97.8
Rotamer outliers (%)	3.6	2.6	1.9

### Analyses of the crystallographic models

The models were analyzed using MolProbity.^22^ The secondary structure was inspected with DSSP^23^ and the search for structural homologues and comparisons were performed with the DALI server.^24^ Analysis of the quaternary structure and the interface surface areas for the two major conformations observed was carried out with PISA.^25^ The average *B*‐factors of each subunit were determined using the program BAVERAGE.^17^ Amino acid sequences were analyzed with Clustal Omega.^26^ Structural alignments and molecular images were prepared using PyMol (www.pymol.org) and ALINE.^27^

## RESULTS AND DISCUSSION

**Figure 1:**
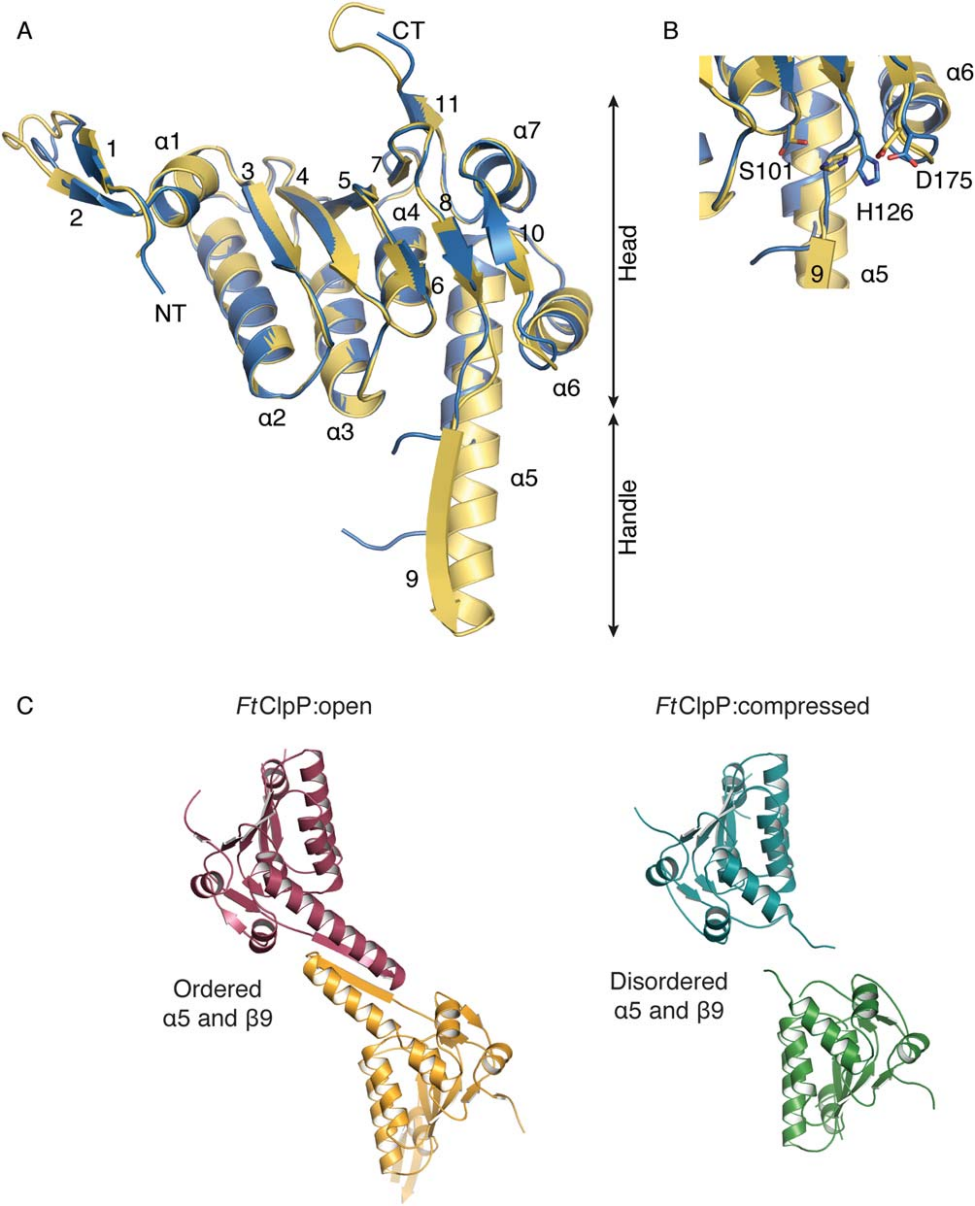
**A.** The ClpP subunit. An overlay ribbon diagram of the open form (yellow) with the closed form (cyan). NT and CT mark the N‐terminal and C‐terminal residues. The assigned secondary structure of *Ft*ClpP is represented as cylinders (α‐helices) and arrows (β‐strands). **B.** A close up view and showing the Ser‐His‐Asp catalytic triad residues in the active sites of open (yellow) and compressed forms (cyan). **C.** A side‐by‐side comparison of a pair of subunits in the open (left) and compressed (right) conformations. Note the order‐disorder difference that involves the handle regions of the subunit at a subunit‐subunit interface.

An efficient *E. coli* expression system and a robust purification protocol for *Ft*ClpP was established that yielded about 10 mg of enzyme per liter of bacterial culture. Analysis by SDS‐PAGE and mass spectrometry (data not shown) indicated that the samples were of high purity and of the correct mass. Size exclusion chromatography, in the presence and absence of 10% glycerol to assist solubility indicated a high level of purity and an oligomer, a single species of approximate mass 290 kDa (Supporting Information Fig. S1A). Subsequently when the sample was applied to a native gel, two species become evident with approximate masses of 160 kDa and 290 kDa (Supporting Information Fig. S1B). This suggests the presence of heptameric and tetradodecameric species in the material used for crystallization experiments. Access to sufficient quantities of material supported a crystallization screen and led to the identification of reproducible crystallization conditions. Three crystal forms of *Ft*ClpP have been characterized and these correspond to a closed or compressed conformation (termed *Ft*ClpP:I) and two structures of an open state (*Ft*ClpP:II and *Ft*ClpP:III), determined at 2.8 Å, 1.9 Å and 1.7 Å resolution, respectively. The structures were solved by molecular replacement (Table 1). Each subunit (comprising an average of 175 residues in the case of the compressed conformation and 190 in the open forms) presents the characteristic ClpP family fold consisting of eleven β‐strands and seven α‐helices [Fig. 1(A)]. In all structures, the subunits are labeled alphabetically. The β‐strands 1 and 2 are not ordered in subunit C of *Ft*ClpP:I or in subunits G, K, N, U from *Ft*ClpP:III. In the majority of the subunits of *Ft*ClpP:II and III, electron density and a chemical environment consistent with the presence of acetate was noted in a pocket close to the catalytic triad. This likely occurs due to the use of an acetate buffer in the crystallization conditions. In subunits E, K, L, and O of *Ft*ClpP:III the electron density was suggestive of some larger entity but we could not unambiguously identify this so acetate was retained in these sites.

**Figure 2:**
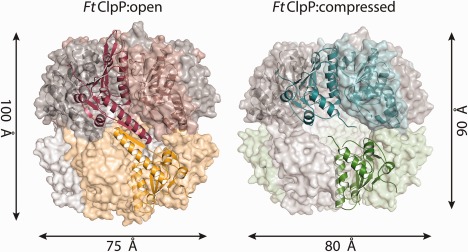
Surface and cartoon representation of the *Ft*ClpP:III (open form; red, yellow and grey, left) and *Ft*ClpP:I (compressed form; green, turquoise and grey, right) tetradecamers. Heptamers are represented with darker colors. A pair of subunits are represented in cartoon ribbon format.

The amino acid sequence of *Ft*ClpP together with the assigned secondary structure is shown in Supporting Information Figure S2. The polypeptide folds into two subdomains, which are termed the head and handle regions [Fig. 1(A)]. The handle is an extension formed by α5 and β9. The active site and catalytic triad, comprising Ser101‐His126‐Asp175, are located in an area between the head and handle regions [Fig. 1(B)]. This is where the link between β6 and α4, the C‐terminal segment of β8 and N‐terminal region of β10 are positioned close to each other.

A search for bacterial ClpP sequences (excluding *Francisella* sp.) in the UniProt database (http://www.uniprot.org/) revealed 405 reviewed/curated entries. These amino acid sequences were aligned and displayed a range of 40–70% identity with *Ft*ClpP extending to strict conservation of the three catalytic residues. Strong conservation of amino acid types was noted in elements of secondary structure with the exception of the C‐terminal region of α5. These features are evident in the amino acid alignment of *Ft*ClpP with three orthologues for which structures have been reported (*Bs*ClpP, *E. coli* ClpP and *M. tuberculosis* ClpP) as depicted in Supporting Information Figure S2. The sequence identity between *Ft*ClpP and *Bs*ClpP is 45% and about 60% with *Ec*ClpP and *Mt*ClpP. Extending from this level of sequence conservation we note a high level of structural similarity is maintained between *Ft*ClpP and *Bs*ClpP (PDB code: 3MT6), *Ec*ClpP (PDB code: 3TT7) and *Mt*ClpP (PDB code: 5DZK) which becomes evident from the least‐squares overlay of all Cα positions in the open form asymmetric units. The r.m.s.d. values are 0.5 ± 0.1, 0.7 ± 0.1, 1.3 ± 0.1 Å, respectively.

*Ft*ClpP forms a heptamer by a lateral association of subunits that involves interaction between β‐strands (3 and 4) and two loops (between β5–β6, and β7–β8) with α‐helices (α2, α3, α4, and α5) of an adjacent subunit. Two heptamers associate to create the tetradecamer exploiting primarily hydrophobic interactions between two α5 helices that are aligned antiparallel to each other, and with hydrogen bonds between the main chain functional groups on a pairing of β9 strands [Fig. 1(C)]. In both *Ft*ClpP:I and II structures, a heptamer constitutes the asymmetric unit and the physiologically relevant tetradecamer is created across a crystallographic two‐fold axis of symmetry. In *Ft*ClpP:III there are 21 subunits in the asymmetric unit and these are arranged to form a complete tetradecamer and one heptamer, which again is positioned at a two‐fold axis that generates another tetradecamer. It is intriguing that the purified sample displayed both heptamer and tetradecamer species when analyzed in a native gel suggesting that distinct conformational states might be present and this may explain why we obtained open and compressed oligomer structures.

There is a high similarity between *Ft*ClpP:II and III, the open form structures. The overlay of Cα positions in the tetradecamers gives an r.m.s.d. of 0.16 Å, so the later is primarily used for further analysis given that it has been determined to a slightly better resolution. In the case of *Ft*ClpP:III, 35 ± 2% of a subunit accessible surface area (ASA) is involved in tetradecamer formation and 43 ± 1% in *Ft*ClpP:I. The buried surface areas for the tetradecamer assemblies are approximately 70% and 55%, respectively. These values indicate the presence of stable macromolecular assemblies^25^ but that in the closed form, *Ft*ClpP:III buries a significantly greater surface area of the oligomeric assembly.

An overlay of Cα positions of individual subunits in *Ft*ClpP:III (r.m.s.d. average of 0.38 ± 0.04 Å using all Cα atoms common to each subunit) indicates a high degree of similarity. The same observation applies to the *Ft*ClpP:I subunits with an average r.m.s.d. 0.21 ± 0.03 Å. Performing pairwise overlays between subunits of *Ft*ClpP:I and *Ft*ClpP:III (r.m.s.d. 0.38 ± 0.04 Å) indicate minor differences, which primarily reside in strands β1 and β2 and at the C‐terminus of the polypeptide. Even when heptamers are compared, there are no major structural differences (r.m.s.d. of 0.78 Å). In Figure 1(A) the overlay of a subunit in each form is presented and the overall close similarity is evident. However, the overlay of the open and closed tetradecamer assemblies results in an r.m.s.d. of 4.29 Å which quantifies the large, and obvious structural difference between open and closed forms of *Ft*ClpP [Figs. 1(C) and 2, Supporting Information Fig. S3].

As in other ClpP structures, it appears that the conformational change between open and closed forms is associated with an order/disorder transition of the “handle” subdomain [Fig. 1(A,C)]. In particular for *Bs*ClpP there is a change driven by the distortion/straightening of α5.^28^ In *Ft*ClpP we observe something different. The handle in the open form, the α5‐β9 segment, is well ordered but in the closed form we did not observe interpretable density with disorder evident. This results in a rearrangement of the subunit positions with respect to each other and in the loss of the active site groove organization [Figs. 1(C) and 2, Supporting Information Fig. S4]). There are changes at the active site, and equatorial pores are created where the reaction products might be released. The consequences of conformational changes involving the handle in *Ft*ClpP however are similar to those previously described, for example, for *Bs*ClpP.^5^, ^28^

## Supporting information

Supporting Information
