# Supplementary material for: Open and compressed conformations of Francisella tularensis ClpP
Source: Proteins. 2016 Nov 20;85(1):188–94. doi: 10.1002/prot.25197 (PMC5225881; doi:10.1002/prot.25197)

Supplementary information

Open and compressed conformations of *Francisella tularensis* ClpP.

Laura Díaz-Sáez, Genady Pankovs and William N. Hunter.

Figure S1. A. Size exclusion chromatography trace for *Ft*ClpP in the presence (cyan) and absence (red) of 10% glycerol. **B.** Calibration of the column based on five standards and positions of peaks in the presence (bottom, cyan dot)) and absence (top, red dot) of 10% glycerol. An arrow marks the theoretical position where a tetradecamer would be observed. **C.** Native gel. Lane 1 Mwt markers. Lane 2 and 3, loadings of 7 and 5 ng protein respectively.

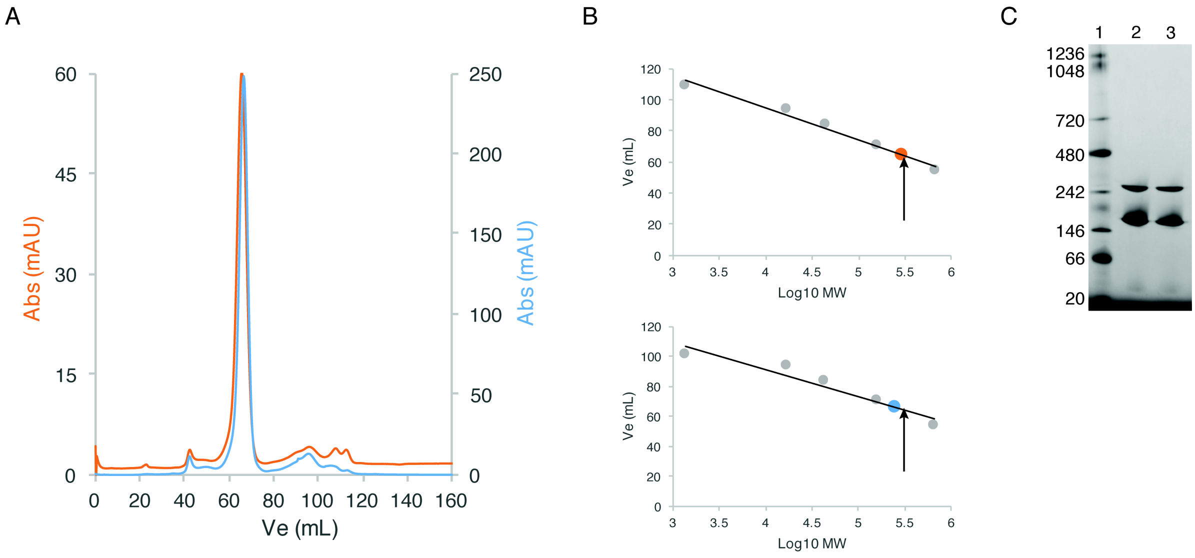

Figure S2. Sequence alignment of *Ft*ClpP (UniProt code Q5NH47), *Bs*ClpP (P80244), *Ec*ClpP (P0A697) and *Mt*ClpP (P9WPC3). The assigned secondary structure of *Ft*ClpP is represented as red cylinders (α-helices) and blue arrows (β-strands). The catalytic triad residues are marked with green triangles.

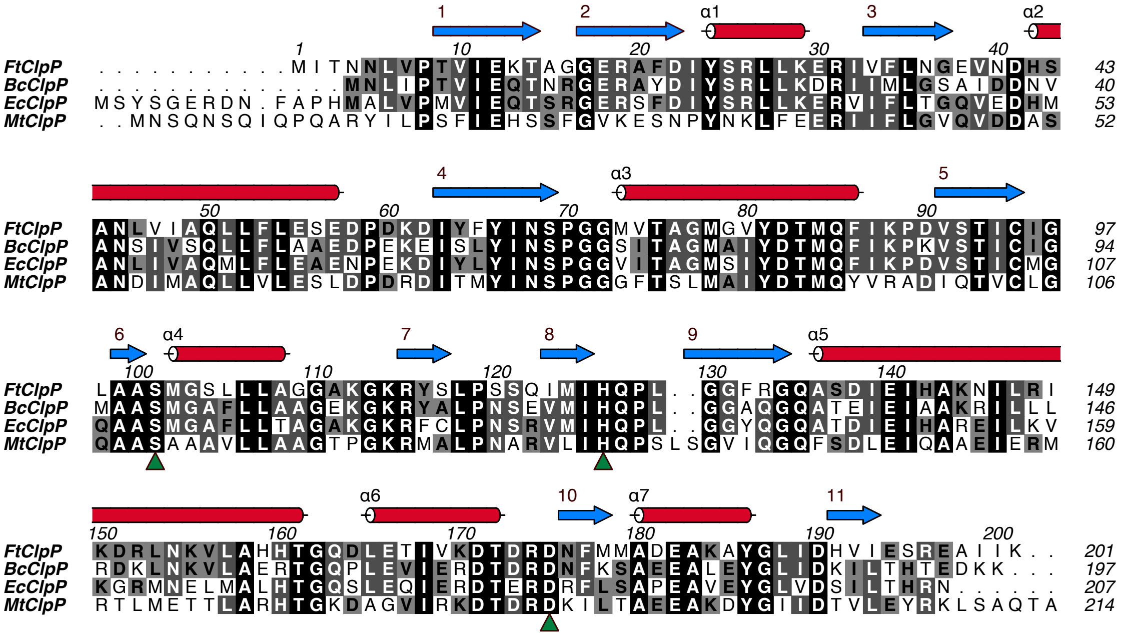

Figure S3. Cartoon representation of the structural overlays of *Ft*ClpP:I (blue) and *Ft*ClpP:III (yellow) monomers **(A)**, heptamers **(B)** and tetradecamers **(C)**. Loops have been simplified in panels B and C for clarity. In panels B and C, side and top views are given.

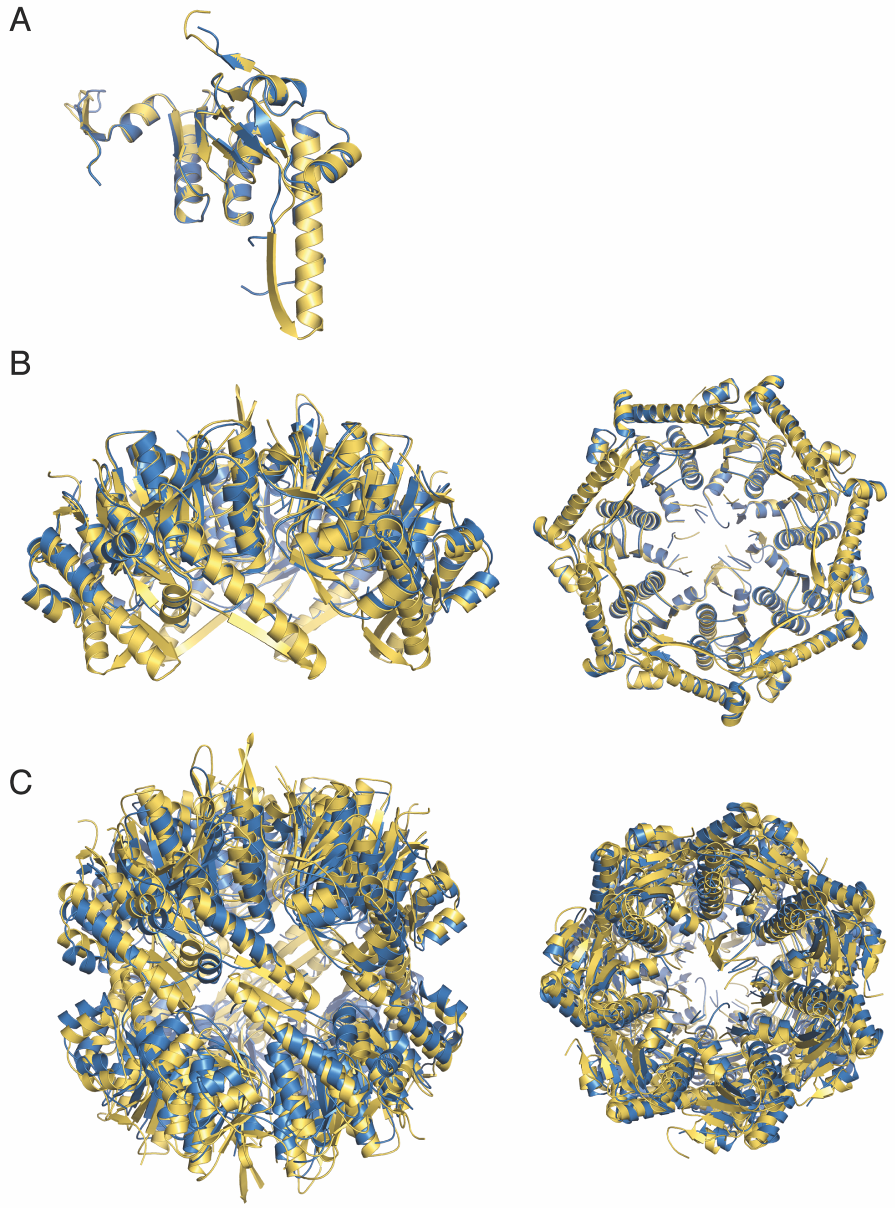

Figure S4. Surface representation of the catalytic chamber created in the tetradecamers. *Ft*ClpP:III (left) coloured in red and grey, and *Ft*ClpP:I (right) in green and grey. Residues that form the catalytic triad are coloured in yellow. The red arrows indicate the active site grooves and blue the equatorial pores.

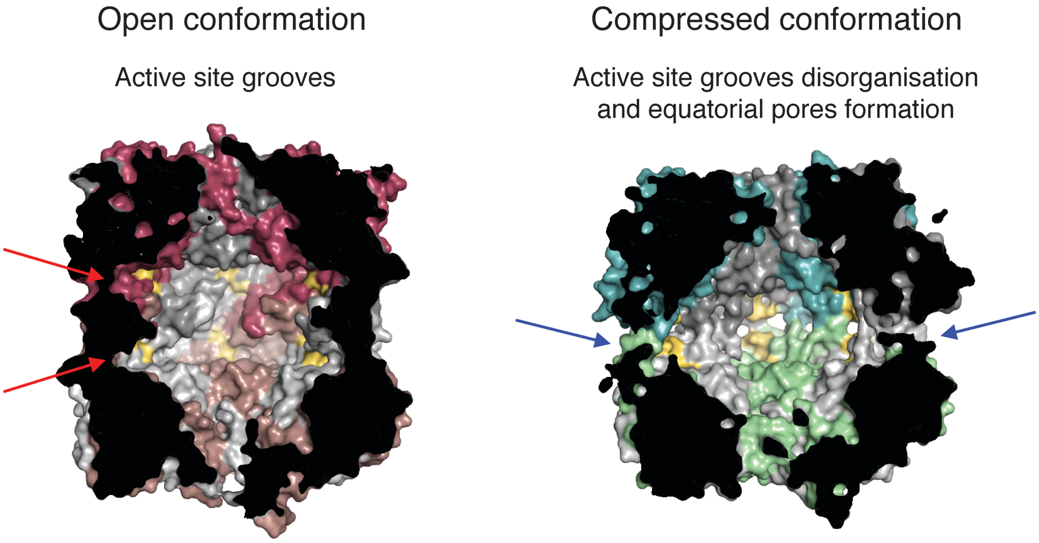

Supplement: Supplementary file 1 — Supporting Information [file PROT-85-188-s001.pdf]
